# Assessment of the influence of selected stress factors on the growth and survival of *Listeria monocytogenes*

**DOI:** 10.1186/s12866-023-02766-4

**Published:** 2023-01-24

**Authors:** Natalia Wiktorczyk-Kapischke, Krzysztof Skowron, Ewa Wałecka-Zacharska, Katarzyna Grudlewska-Buda, Kacper Wnuk, Katarzyna Buszko, Eugenia Gospodarek-Komkowska

**Affiliations:** 1 Department of Microbiology, Ludwik Rydygier Collegium Medicum in Bydgoszcz, Nicolaus Copernicus University, Toruń, Poland; 2 Department of Food Hygiene and Consumer Health, Wrocław University of Environmental and Life Sciences, Wrocław, Poland; 3 Department of Theoretical Foundations of Biomedical Sciences and Medical Computer Science, Ludwik Rydygier Collegium Medium in Bydgoszcz, Nicolaus Copernicus University, Toruń, Poland

**Keywords:** *Listeria monocytogenes*, Acid stress, Heat stress, Nutrition stress, Osmotic stress, Cold stress, Freezing-defrosting, Stress factors

## Abstract

**Background:**

*Listeria monocytogenes* are Gram-positive rods, which are the etiological factor of *listeriosis*. *L. monocytogenes* quickly adapts to changing environmental conditions. Since the main source of rods is food, its elimination from the production line is a priority. The study aimed to evaluate the influence of selected stress factors on the growth and survival of *L. monocytogenes* strains isolated from food products and clinical material.

**Results:**

We distinguished fifty genetically different strains of *L. monocytogenes* (PFGE method). Sixty-two percent of the tested strains represented 1/2a-3a serogroup. Sixty percent of the rods possessed ten examined virulence genes (*fbpA*, *plcA*, *hlyA*, *plcB*, *inlB*, *actA*, *iap*, *inlA*, *mpl, prfA*). Listeria Pathogenicity Island 1 (LIPI-1) was demonstrated among 38 (76.0%) strains. Majority (92.0%) of strains (46) were sensitive to all examined antibiotics. The most effective concentration of bacteriophage (inhibiting the growth of 22 strains; 44.0%) was 5 × 10^8^ PFU. In turn, the concentration of 8% of NaCl was enough to inhibit the growth of 31 strains (62.0%). The clinical strain tolerated the broadest pH range (3 to 10). Five strains survived the 60-min exposure to 70˚C, whereas all were alive at each time stage of the cold stress experiment. During the stress of cyclic freezing-defrosting, an increase in the number of bacteria was shown after the first cycle, and a decrease was only observed after cycle 3. The least sensitive to low nutrients content were strains isolated from frozen food. The high BHI concentration promoted the growth of all groups.

**Conclusions:**

Data on survival in stress conditions can form the basis for one of the hypotheses explaining the formation of persistent strains. Such studies are also helpful for planning appropriate hygiene strategies within the food industry.

## Background

*Listeria monocytogenes* are Gram-positive, facultatively anaerobic, non-spore-forming rods. Bacteria settle many niches due to their tolerance to changing environmental conditions (natural, food processing or the host organism environment) [1]. Major source of the pathogen is food (dairy, meat and fish products, raw vegetables and fruits, RTE (ready-to-eat) products). *L. monocytogenes* infections (*listeriosis)* are characterized by a high mortality rate (up to 30%) [2]. There have been many recorded outbreaks of *listeriosis* related to various food products (including Enoki Mushroooms, frozen vegetables, raw milk, cantaloupes) [3], including the deadliest one in the Republic of South Africa in 2017–2018 (ready-to-eat processed meat products, 200 deaths) [4].

During food processing, *L. monocytogenes* experiences many unfavorable conditions, including low and high temperatures, variable pH, osmotic stress, nutrient deficiency, and contact with disinfectants. The most commonly encountered stresses within the food industry for *L. monocytogenes* are acidic and osmotic stresses (e.g., fruit with low pH, processes of drying sausages, brines), temperature (e.g., pasteurization) [5]. The ability of *L. monocytogenes* to adapt to low pH environments, e.g., during the passage through the stomach and in the phagosome, is relevant for the host invasion [6]. The adaptive stress response of *L. monocytogenes* is indispensable for the survival in the food environment and may affect bacterial pathogenicity [7]. One of the main stress response mechanisms is associated with alternative sigma factors: σ^B^, σ^C^, σ^H^ and σ^L^, which σ^B^ plays a key role [8]. In *L. monocytogenes*, these factors control more than 300 genes, including stress and virulence genes. The use of sublethal doses of stress factors in the food processing environment (FPE) may increase resistance to antibiotics or stimulate resistance to other stress conditions (cross-resistance) and higher doses of the same stress (stress adaptation) [9].

In recent years a growing concern has focused on the identification of strains that survive in the food processing environment [10–12]. Researchers have suggested that multiple isolations of the same strain over a period of time indicate the appearance of persistent strains [13]. One of the main theories for the emergence of persistent strains is resistance to stress factors [14]. The ability to adapt to the encountered stress factors contributes to the extended survival within one environment, leading to cross-contamination of food products. It is worth noting that the cases of listeriosis are more and more often associated with drug-resistant strains [15].

Microbiological typing (also known as "fingerprinting" or "characterization") is a tool that has been used for years to identify organisms within a species [16, 17].There are several methods for typing microorganisms. Typing microorganisms can be used, e.g., for epidemiological purposes (source of the epidemic) [18–21], food safety [22], as well as to identify strains that have persisted in the environment [12, 23–25]. Currently, Pulsed-field gel electrophoresis (PFGE) is a”gold standard” for typing bacteria [26]. Other methods of genetic typing include among others: Random Amplified Polymorphic DNA (RAPD) [27, 28], Restriction Fragments Length Polymorphism (RFLP) [29], Amplified Fragment Length Polymorphism (AFLP) [30], whole genome sequencing (WGS), Multi-Locus Sequence Typing (MLST) and matrix-assisted laser desorption/ionization time of flight mass spectrometry (MALDI-TOF MS) [31, 32].

The study aimed to evaluate the impact of selected stress factors (high and low temperatures, cyclic freezing and defrosting, pH (3–10), salinity (0–20%), availability of nutrients) on the growth and survival (screening) of *L. monocytogenes* isolated from food products and clinical material.

## Results

### Evaluation of genetic similarity (pulsed-field gel electrophoresis)

**Fig. 1:**
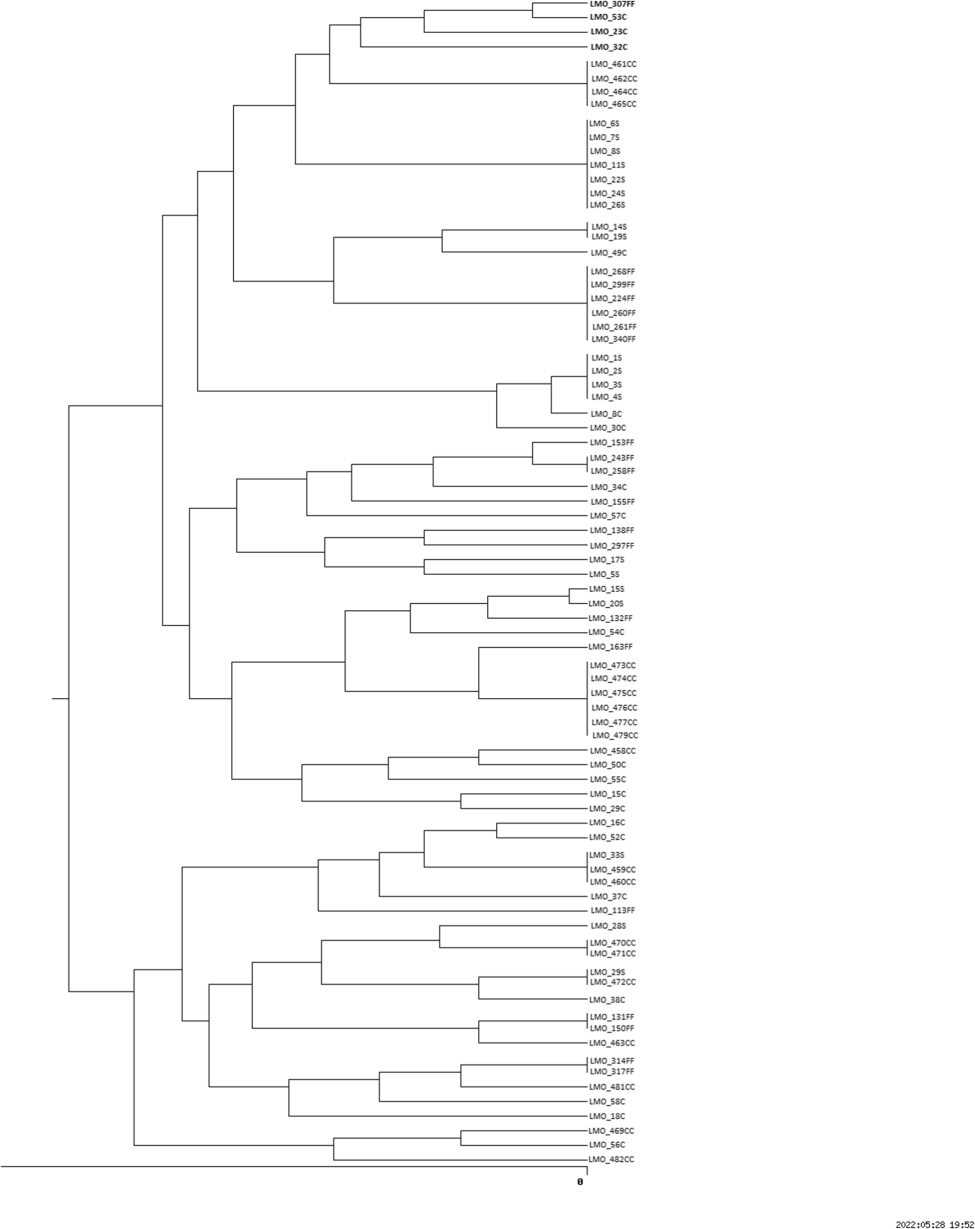
Dendrogram of genetic similarity of isolates (C – clinical, S – salmon, CC- cold cuts, FF- frozen food)

Among the 80 examineed isolates, we have identified 50 genetically different strains of *L. monocytogenes* (Fig. 1), which were used in the further part of the study. Twenty strains derived from clinical material 8, 10 and 12 strains from salmon, cold cuts and frozen food, respectively. The cut-off level was set at 80.0%. We distinguished 12 PFGE profiles: I (461CC, 462CC, 464CC, 465 CC), II (6S, 7S, 8S, 11S, 22S, 24S, 26S), III (14S, 19S), IV (268FF, 299FF, 224FF, 260FF, 261FF, 340FF), V (1S, 2S, 3S, 4S), VI (243FF, 258FF), VII (473CC, 474CC, 475CC, 576CC, 477CC, 479CC), VIII (33S, 459CC, 460CC), IX (470CC, 471CC), X (29S, 472CC), XI (131FF, 150FF), XII (314FF, 317FF) (Fig. 1). We observed genetically identical strains isolated from different sources. Set VIII contained one isolate from salmon and two isolates from cold cuts, and set X the salmon isolate and cold cuts isolate (Fig. 1).

### Preliminary characterization of strains

#### Evaluation of *L. monocytogenes* strains serogroup affiliation

The most studied strains represented the serogroup 1/2a-3a (31 (62.0%)), followed by the serogroup 4b-4d-4e (14 (28.0%)). Most strains of seogroup 1/2a-3a were of clinical origin, while 4b-4d-4e strains derived from frozen food (Table 1). However, based on Fisher’s exact test, there is no association between serogroup affiliation and strain origin (*p* = 0.143).

**Table 1:** Serogroup affiliation of the *L. monocytogenes* strains studied

Serogroup	Clinical (*n* = 20)	Salmon (*n* = 8)	Cold cuts (*n* = 10)	Frozen food (*n* = 12)	Total (*n* = 50)
1/2a-3a	14 (70.0%)	7 (87.5%)	5 (50.0%)	5 (41.7%)	31 (62.0%)
1/2b-3b	1 (5.0%)	0 (0.0%)	2 (20.0%)	0 (0.0%)	3 (6.0%)
1/2c-3c	1 (5.0%)	0 (0.0%)	1 (10.0%)	0 (0.0%)	2 (3.3%)
4b-4d-4c	4 (20.0%)	1 (12.5%)	2 (20.0%)	7 (58.3%)	14 (28.0%)

#### Detection of selected virulence genes

We distinguished four profiles of virulence genes (Table 2), of which the most numerous (60.0% of strains) was profile I (presence of 10 virulence genes). We demonstrated the presence of Listeria Pathogenicity Island 1 (LIPI-1) (genes: *prfA, plcA, hlyA, mpl, actA, plcB*) among 38 (76.0%) tested strains. We did not identify the *mpl* gene in 5 strains and the *fbpA* gene in 8 strains. Seven tested strains representing all groups of isolates possessed 8 virulence genes (profile IV) (Table 2). There was a significant association between gene profiles and strain origin (Fisher’s exact test: p = 0.007).

**Table 2:** Profiles of virulence genes among *L. monocytogenes* strains

Profile	Genes coding for virulence factors	Clinical (*n* = 20)	Salmon (*n* = 8)	Cold cuts (*n* = 10)	Frozen food (*n* = 12)	Total (*n* = 50)
I	*fbpA, hlyA, plcA, actA, inlB, plcB, iap, inlA, mpl, prfA*	15 (75.0%)	6 (75.0%)	4 (40.0%)	5 (41.7%)	30 (60.0%)
II	*fbpA, hlyA, plcA, actA, inlB, plcB, iap, inlA, prfA*	4 (20.0%)	1 (12.5%)	0 (0.0%)	0 (0.0%)	5 (10.0%)
III	*hlyA, plcA, actA, inlB, plcB, iap, inlA, mpl, prfA*	0 (0.0%)	0 (0.0%)	4 (40.0%)	4 (33.3%)	8 (16.0%)
IV	*hlyA, plcA, actA, inlB, plcB, iap, inlA, prfA*	1 (5.0%)	1 (12.5%)	2 (20.0%)	3 (25.0%)	7 (14.0%)

#### Evaluation of drug resistance

We distinguished three antibiotic sensitivity profiles. The most numerous profile (A) included 46 (92.0%) tested strains sensitive to all tested antibiotics (Table 3). Resistance to penicillin was demonstrated in three clinical strains (profile B), and resistance to erythromycin in one strain isolated from salmon (Table 3). Penicillin-resistant strains belonged to serogroups: 1/2a-3a, 1/2c-3c, 4b-4d-4e, and the erythromycin-resistant strain represented serogroup 1/2a-3a (Table 4). There was no relationship between drug-resistance profiles and origin of strains (Fisher’s exact test: *p* = 0.180).

**Table 3:** Drug-resistance profiles of *L. monocytogens* strains

Profile	Antibiotics	Clinical (*n* = 20)	Salmon (*n* = 8)	Cold cuts (*n* = 10)	Frozen food (*n* = 12)	Total (*n* = 50)
A	R:-; S: P, AMP, MEM, E, STX	17 (85.0%)	7 (87.5%)	10 (100.0%)	12 (100.0%)	46 (92.0%)
B	R: P; S: AMP, MEM, E, STX	3 (15.0%)	0 (0.0%)	0 (0.0%)	0 (0.0%)	3 (6.0%)
C	R: E; S: P, AMP, MEM, STX	0 (0.0%)	1 (12.5%)	0 (0.0%)	0 (0.0%)	1 (2.0%)

*R* resistance, *S* sensitive, *P* penicillin, *AMP* ampicillin, *MEM* meropenem, *E* erythromycin, STX co-trimoxazole (sulfamethoxazole + trimethoprim)

**Table 4:** Drug resistance of *L. monocytogenes* strains by serogroup

Antibiotics	Number of strains by origin			
Serogroup	1/2a-3a	1/2b-3b	1/2c-3c	4b-4d-4e
**sensitive to all tested antibiotics**	13C, 6S, 5CC, 5FF	1C, 2CC	1CC	3C, 1S, 2CC, 7FF
**resistance to penicillin**	1C	-	1C	1C
**resistance to erythromycin**	1S	-	-	-

*C* clinical, *S* salmon, *CC* cold cuts, *FF* frozen food

### Evaluation of minimum inhibitory concentration (MIC) and minimum bactericidal concentration (MBC) of Phage Guard L

The concentration of PhageGuard L in the range of 5 × 10^4^ to 5 × 10^5^ PFU (Plaque Forming Units) did not inhibit the growth of the tested *L. monocytogees* strains (based on visually determined turbidity of the broth). The concentration of 5 × 10^6^ PFU inhibited the growth of one strain (isolated from clinical material), while 5 × 10^7^ PFU—5 strains (10.0%) (no observed turbidity). The most effective concentration of bacteriophage inhibiting the growth of the largest number of strains (22; 44.0%) was 5 × 10^8^ PFU. The concentration of 5 × 10^10^ PFU inhibited the growth of 11 strains (22.0%). One strain isolated from salmon and two obtained from frozen food were insensitive to all studied concentrations of PhageGuard L (visible turbidity) (Table 5).

**Table 5:** Minimum Inhibitory Concentration and Minimum Bactericidal Concentration value of Phage Guard L

Strain group	MIC value [PFU]						MBC value [PFU]			
	5 × 10^6^	5 × 10^7^	5 × 10^8^	5 × 10^9^	5 × 10^10^	Above 5 × 10^10^	5 × 10^8^	5 × 10^9^	Growth in 5 × 10^10^	No impact
**Clinical** **(*n* = 20)**	1 (5.0%)	0 (0.0%)	10 (50.0%)	2 (10.0%)	7 (35.0%)	0 (0.0%)	3 (15.0%)	1 (5.0%)	16 (80.0%)	0 (0.0%)
**Salmon** **(*n* = 8)**	0 (0.0%)	4 (50.0%)	3 (37.5%)	0 (0.0%)	0 (0.0%)	1 (12.5%)	0 (0.0%)	0 (0.0%)	7 (87.5%)	1 (12.5%)
**Cold cuts** **(*n* = 10)**	0 (0.0%)	0 (0.0%)	4 (40.0%)	4 (40.0%)	2 (20.0%)	0 (0.0%)	0 (0.0%)	0 (0.0%)	10 (100.0%)	0 (0.0%)
**Frozen food** **(*n* = 12)**	0 (0.0%)	1 (8.3%)	5 (41.7%)	2 (16.7%)	2 (16.7%)	2 (16.7%)	1 (8.3%)	0 (0.0%)	9 (75.0%)	2 (16.7%)
**Total (*n* = 50)**	1 (2.0%)	5 (10.0%)	22 (44.0%)	8 (16.0%)	11 (22.0%)	3 (6.0%)	4 (8.0%)	1 (2.0%)	42 (84.0%)	3 (6.0%)

*MIC* Minimum Inhibitory Concentration, *MBC* Minimum Bactericidal Concentration, *PFU* Plaque Forming Units

The MBC (no observed growth on solid medium) value of PhageGuard L was – 5 × 10^8^ PFU and 5 × 10^9^ PFU, for 5 and 1 strain, respectively (Table 5). For the other strains, the bactericidal effect of PhageGuard L was not demonstrated in the tested concentration range.

### Influence of selected stress factors on *L. monocytogenes*

#### Osmotic stress

**Fig. 2:**
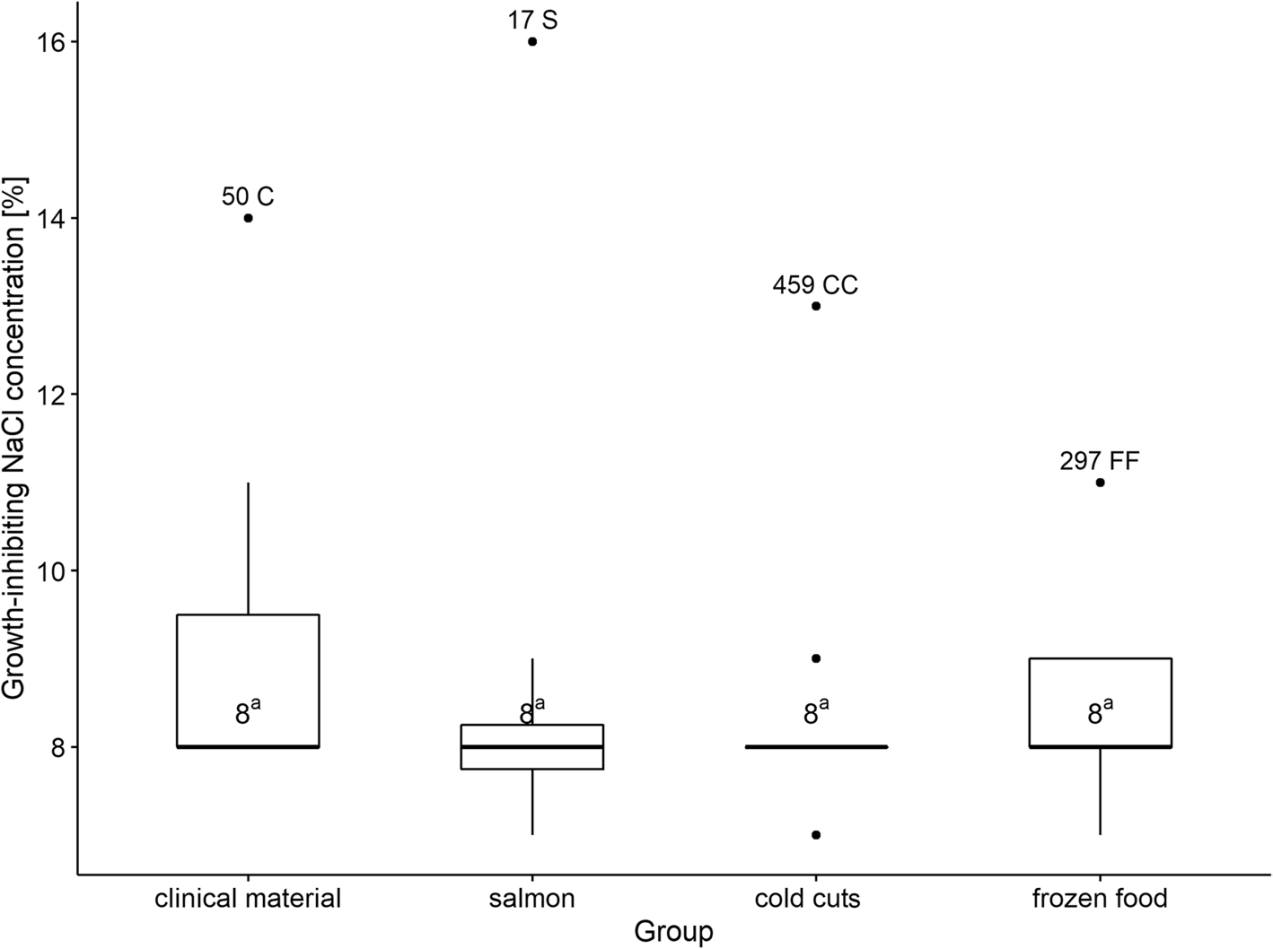
Comparison of strain groups (origin) based on salinity. Boxplot: centre: median, box limits: 25th and 75th centiles, whiskers extend to the smallest/largest value no further than median ± 1.5 times interquartile range (IQR). (C – clinical, S – salmon, CC – cold cuts, FF – frozen food); a,b—values marked with different letters differ in a statistically significant way

The lowest concentration inhibiting the growth of the tested strains was 7% NaCl, at this concentration growth inhibition was observed for 5 out of 50 strains (10.0%). The concentration of 8% NaCl was enough to inhibit the growth of strains 31 (62.0%). Then, the concentration of 9% NaCl inhibited the growth of 6 more examined strains (12.0%), and 11% NaCl—5 (10.0%) strains. The concentration of 13% NaCl inhibited the growth of one strain isolated from cold cuts (459CC), and 14% NaCl—one strain isolated from the clinical material (50C) (Fig. 2).

The median NaCl concentration tolerated by strains from all groups (clinical material, salmon, cold cuts, frozen food) was 8% (Fig. 2). The highest concentration tolerated by one strain (no. 17S), isolated from salmon, was 15%. In the other groups the highest NaCl concentration enabling the survival was 10%, 12%, 13% for frozen food (no. 297FF), cold cuts (no. 459CC) and clinical material strain (no. 50C), respectively. There were no significant differences between strain groups. The most resistant strains are labeled on boxplots in Fig. 2.

#### Acid and alkaline stress

The study assessed the pH in the range of 3 to 10. The analysis of the results included “the growth-inhibiting pH value < 7” and “the growth-inhibiting pH value > 7”. When growth (visible turbidity in the broth, in triplicate) was found at pH 10 and pH 3, there were arbitrarily assinged values 11 and 2 as growth-inhibiting, respectively.

**Fig. 3:**
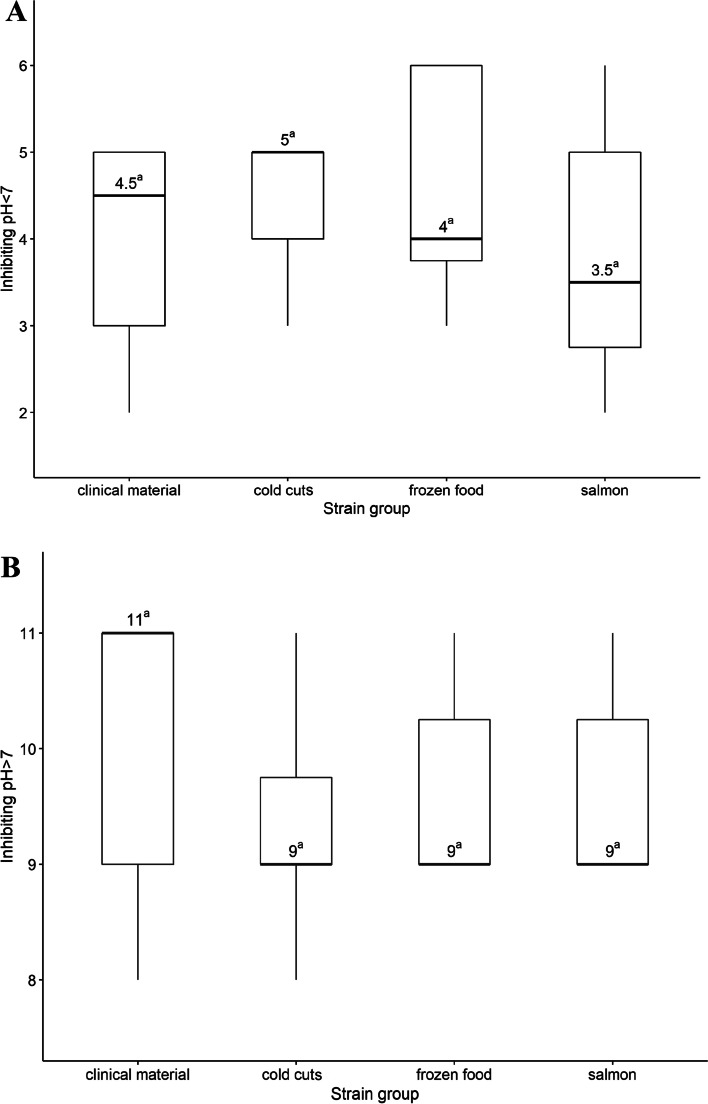
Comparison of strain groups based on (**A**) the lowest (**B**) the highest pH that inhibited the growth of particular strain. Boxplot: centre: median, box limits: 25th and 75th centiles, whiskers: minimum and maximum. a,b—values marked with different letters differ in a statistically significant way

The median for "the growth-inhibiting pH value < 7" was 4.5, 5, 4 and 3.5 for strains isolated from clinical matrerial, cold cuts, frozen food and salmon, respectively. In contrast, the median in “the growth-inhbiting pH value > 7” variant was 11, 9, 9, and 9 for strains isolated from the clinical matrial, cold cuts, frozen food and salmon, respectively (Fig. 3).

In "the growth-inhibiting pH value < 7" variant, pH 6 inhibited 5 (10.0%) strains. Further lowering of pH resulted in the inhibition of additional 19 (38.0%) strains – pH 5, 12 (24.0%) strains – pH 4, and 11 (22.0%) strains – pH 3. At pH 7, the growth of all tested strains was observed. In "the growth-inhbiting pH value > 7" variant, pH 8 inhibited the growth of 6 (12.0%) strains, pH 9 – additional 20 (40.0%) strains, and pH 10 – additional 6 (12.0%) strains. Importantly, 18 strains (36.0%), including 12 strains isolated from the clinical material, survived the pH 10 (Fig. 3). In turn, 3 strains (including two isolated from salmon (no. 15S, 17S) and one from the clinical material (no. 18C), resisted pH 3. The source of strain did not affect the acid or alkaline resistance of bacteria.

**Fig. 4:**
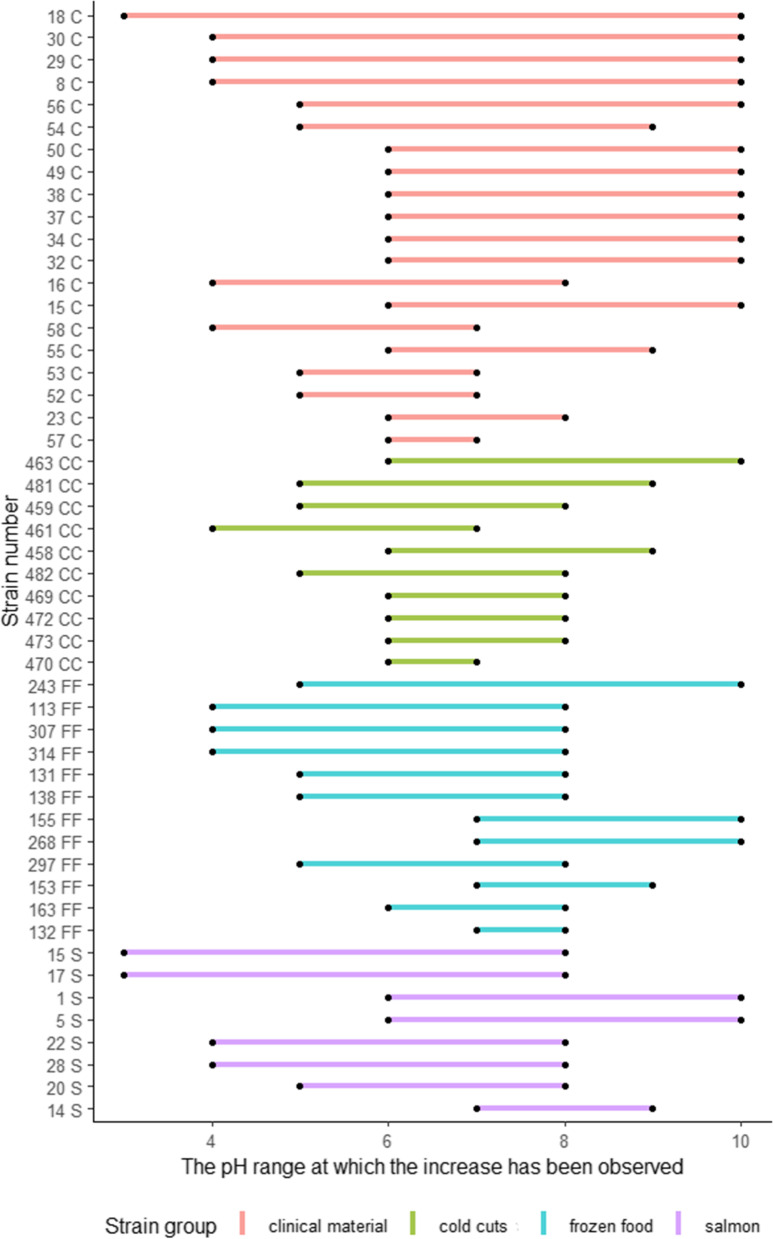
The pH range at which the growth has been observed (C – clinical, S – salmon, CC – cold cuts, FF- frozen food)

Ten strains with the growth inhibited at pH 5, tolerated up to pH 10 (better adaptation to alkaline pH). In turn, strains with growth inhibition at pH 3, were able to multiply at maximum of pH 8 (Fig. 4). Two strains isolated from salmon survived in a broad range of acid stress (pH 2—8) (Fig. 4).

The clinical strain (no. 18C, range 3–10) tolerated the widest pH range (visible turbidity in the broth, in triplicate), while three strains were able to grow only in the very narrow range of pH (range 6–7 for strains No. 58C, 470CC and the range 7–8 for strain No. 132FF) (Fig. 4).

#### Heat and cold stress

**Fig. 5:**
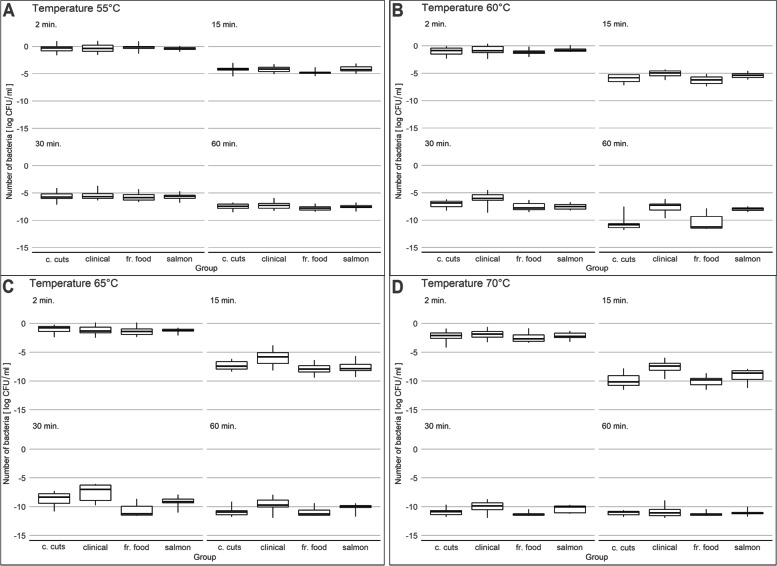
Number of bacteria observed for individual combinations of time points and temperatures for each strain group. Boxplot: centre: median, box limits: 25th and 75th centiles, whiskers: minimum and maximum. **A** 55˚C, **B** 60˚C, **C** 65˚C, **D** 70˚C

We demonstrated the growth of the tested strains at 55˚C, 60˚C (2, 15, 30 and 60 min) and 65˚C (2, 15 min). Exposure to 55˚C for 60 min did not inhibit the growth of the tested *L. monocytogenes* strains (median: 3.72 log CFU/ml). The lowest growth-inhibiting temperature in the study was 65˚C, the exposure time was 30 min. In this variant, we did not demonstrate survival of 10 strains, including 8 frozen food (no. 113FF, 131FF, 132FF, 153FF, 155FF, 163FF, 243FF, 307FF), one salmon (no. 20S) and one cold cuts (no. 463CC) strains. The 60-min exposureof 65˚C inhibited the growth of 16 tested strains (8 from cold cuts and 8 from frozen food). Five strains, including 4 isolated from clinical material (no. 58C, 57C, 56C, 55C) and one isolated from salmon (no. 17S) (Fig. 5) survived exposure to 70˚C for 60 min.

All strains survived all stages of the cold stress experiment. In the control variant, without the stress factor, the median number of bacteria was as follows: clinical – 6.97 log CFU/ml; salmon—6.68 log CFU/ml; cold cuts—6.34 log CFU/ml; frozen food—6.79 log CFU/ml. After 24 h, the number of CFU increased, median for clinical—7.78 log CFU/ml; salmon – 8.13 log CFU/ml; cold cuts—7.99 log CFU/ml; frozen food – 8.01 log CFU/ml. After 30 days of the experiment, a significant increase in the number of CFUs of bacteria was visible, median for clinical—11.02 log CFU/ml; salmon—10.42 log CFU/ml; cold cuts—10.12 log CFU/ml; frozen food—10.12 log CFU/ml.

**Fig. 6:**
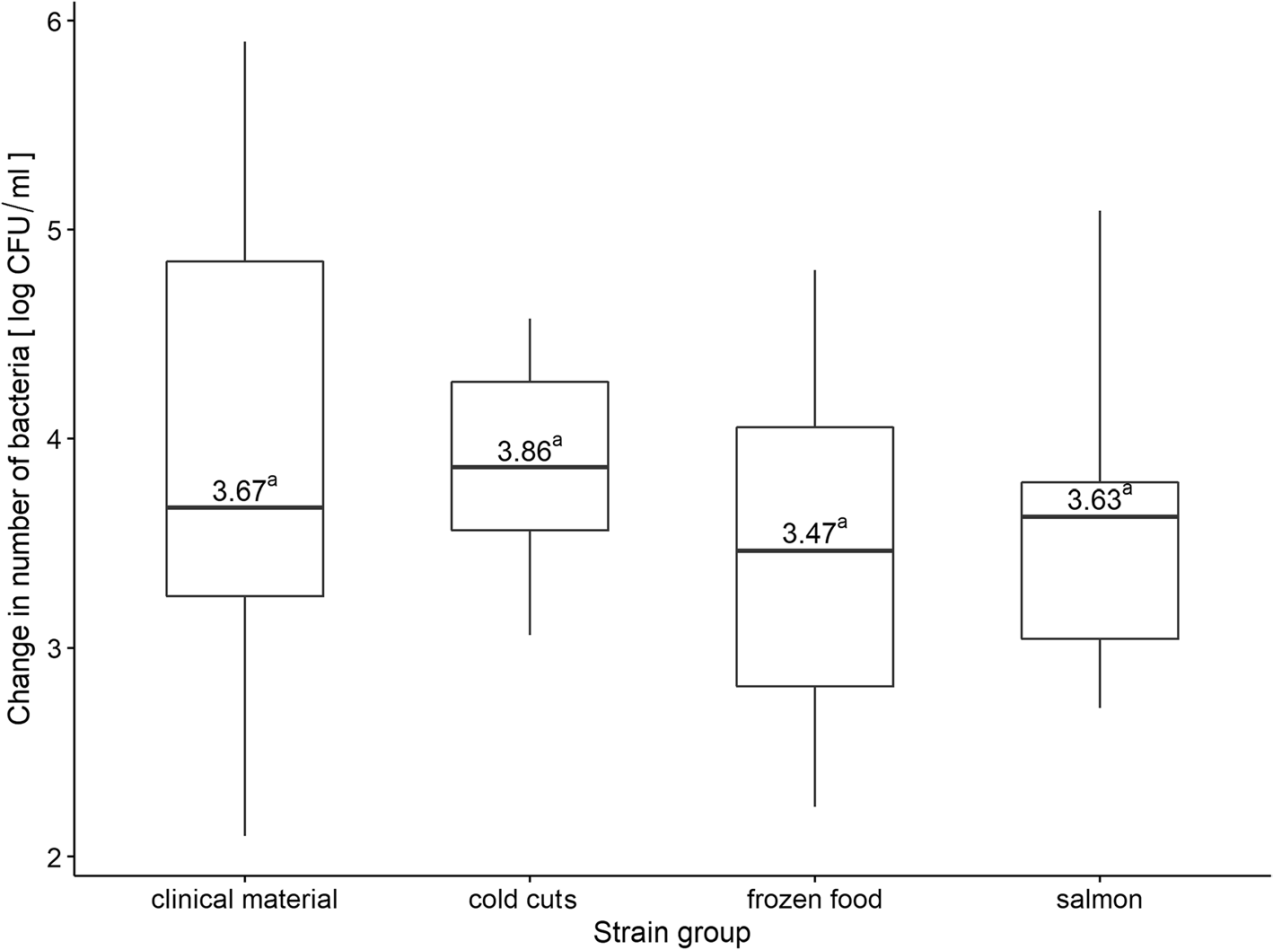
Change in the number of bacteria (relative to control variant) after exposure to cold stress in relation to the groups of origin of the strains. Boxplot: centre: median, box limits: 25th and 75th centiles, whiskers: minimum and maximum. a,b—values marked with different letters differ in a statistically significant way (*p* ≤ 0.05)

In order to compare the response to cold stress, the difference between the number of bacteria after 30 days and the number of control bacteria (without stress, day 0) was examined for each group; however, there were no significant differences (Fig. 6).

#### The effect of cyclic freezing and defrosting

**Fig. 7:**
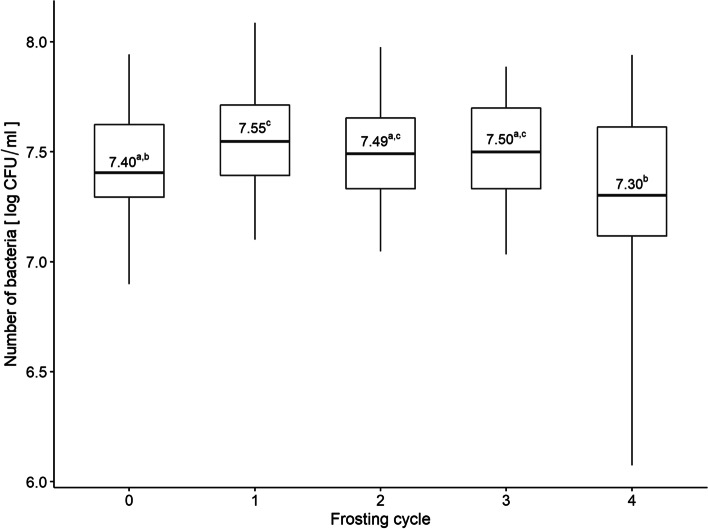
Comparison of subsequent cycles of frosting and defrosting based on number of bacteria. Boxplot: centre: median, box limits: 25th and 75th centiles, whiskers: minimum and maximum. a,b,c – differences between values marked with different letters are statistically significant

The median number of bacteria (without the stress factor) was 7.40 log CFU/ml. Thereafter, the median number of bacteria was 7.55, 7.49, 7.50 and 7.30 log CFU/ml after 1, 2, 3, 4 freezing-defrosting cycles, respectively. In the variant with defrosting only after the 4th cycle, the median number of bacteria was 7.44 log CFU/ml (Fig. 7).

The Wilcoxon test showed significant differences between the cycles 0 and 1 (a visible increase in the number of bacteria), and also between cycles 4 and 1, 2, 3 (after the fourth cycle the number of bacteria decreased) (Fig. 7). There were no significant differences (*p* = 0.074) in the bacteria number between the variant of 4 cycles of freezing-defrosting and the variant of defrosting after the fourth cycle.

#### Influence of nutrients

To comapre the groups of strains, we divided the results according to the nutrient content: low (10–40%) and high (130–200%) BHI content. The results are presented as the change (relative to control) in the number of bacteria (an increase in the number of bacteria with an increase in nutrient content).

We assessed the impact of low and high availability of nutrients on *L. monocytogenes* growth. In each experiment variant, the growth of the tested strains was observed. The median bacterial count for the 10% nutrient availability variant, was: 6.11, 6.18, 5.59, 6.65 log CFU/ml for clinical, salmon, cold cuts and frozen food strains, respectively. In turn, in the variant with the highest availability of nutrients (200%), the median number of bacteria was: 13.30, 13.51, 13.23, 12.26 log CFU/ml, for clinical, salmon, cold cuts and frozen food strains, respectively.

**Fig. 8:**
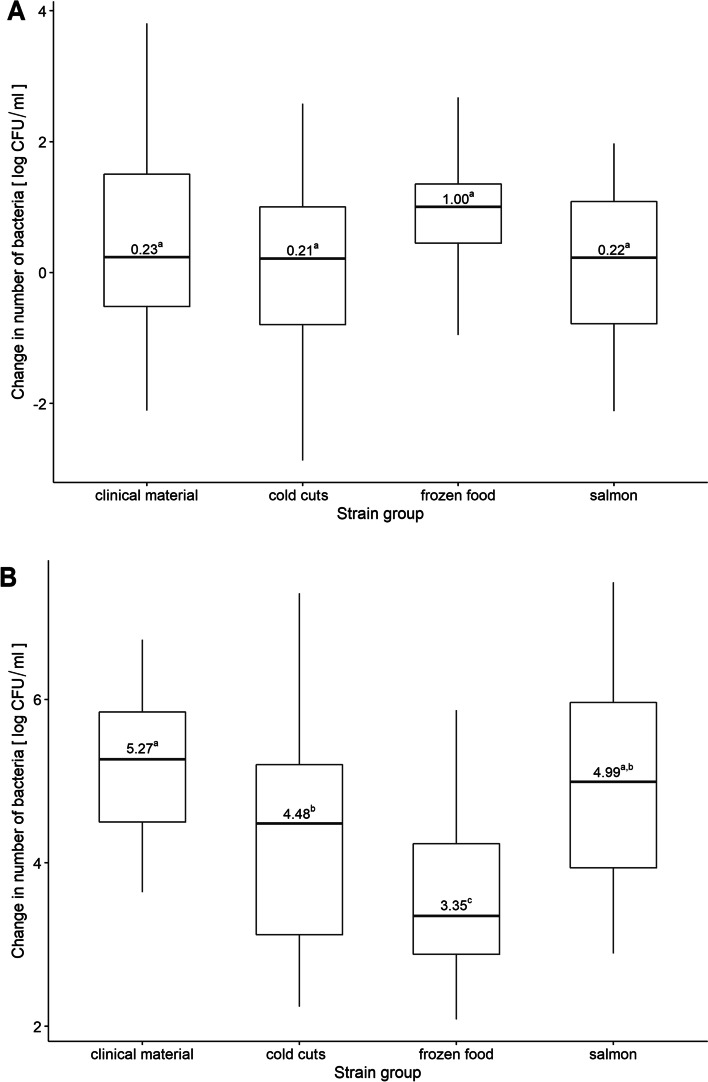
Boxplots for (**A**) low and (**B**) high dose of BHI for each group. Boxplot: centre: median, box limits: 25th and 75th centiles, whiskers: minimum and maximum. a,b—values marked with different letters differ in a statistically significant way

In the low BHI (Brain Heart Infusion) (10–40%) variant, the median number of bacteria was 0.23, 0.21, 0.22 and 1.0 log CFU/ml for strains isolated from clinical material, cold cuts, salmon and frozen food, respectively. The least sensitive to low BHI content were strains isolated from frozen food. However, we did not observe no significant differences between the groups of strains. In the high BHI concentration (130–200%) variant, the median number of bacteria was 5.27, 4.48, 3.35 and 4.99 log CFU/ml for strains isolated from the clinical material, cold cuts, frozen food and salmon, respectively (Fig. 8). The high BHI content promoted the growth of all groups.

#### Ranking of responses to stress factors

**Fig. 9:**
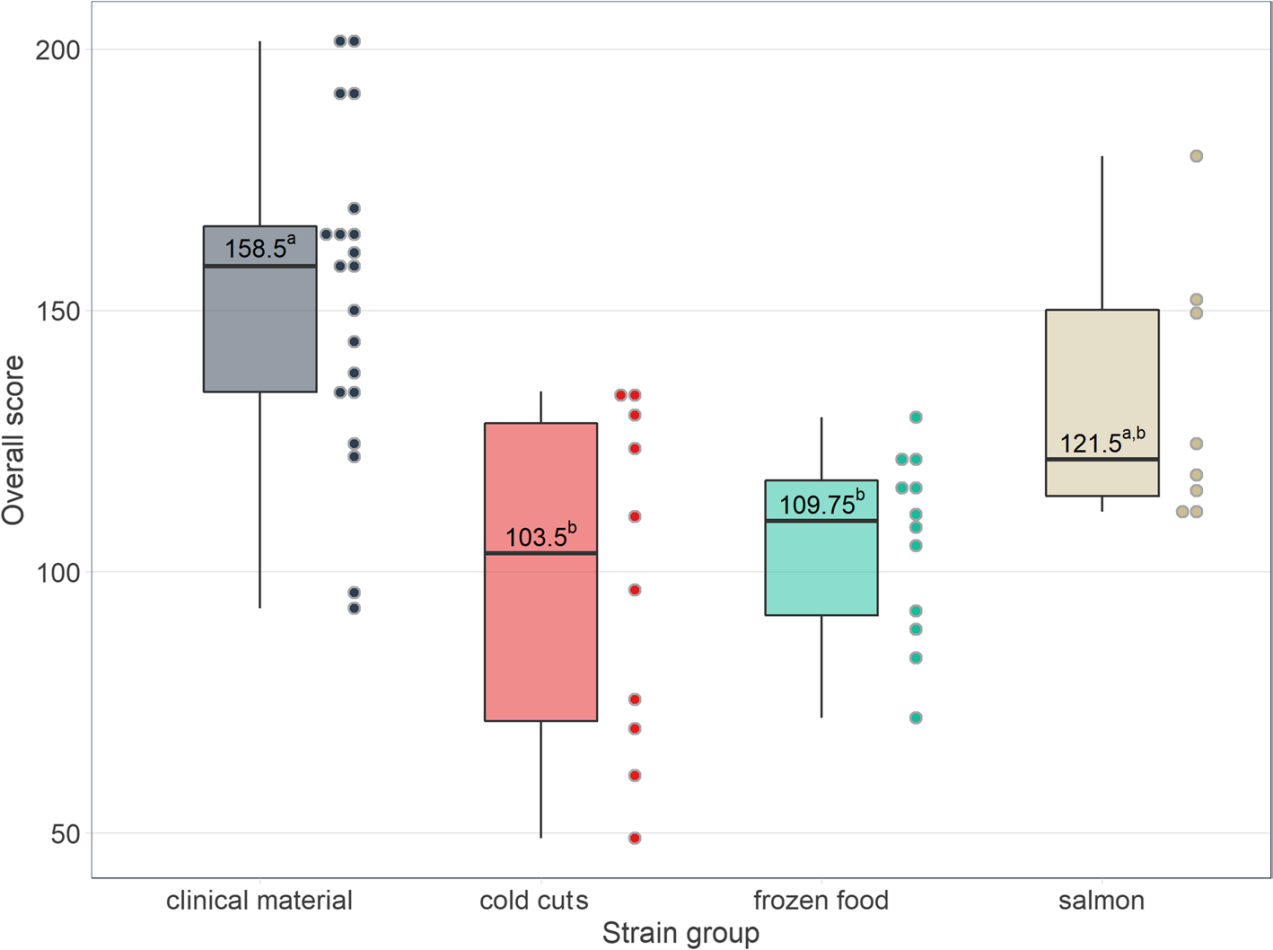
Overall score for each strain group. The plot contains boxplots (centre: median, box limits: 25th and 75th centiles, whiskers: minimum and maximum) with distribution of data as dots. a,b—values marked with different letters differ in a statistically significant way

The strains were ranked according to the response to the tested stress conditions. The mean score was 158.5, 121.5, 109.75 and 103.5 for strains isolated from clinical material, salmon, frozen food and cold cuts, respectively (Fig. 9). Clinical strains responded to stress conditions the most effectively, whereas strains from cold cuts and frozen food were the least resistant. The highest score received four strains isolated from clinical material and one isolated from salmon (no. 55C, 58C, 56C, 38C, 17S). The most sensitive strains, with the lowest score, were 4 strains isolated from cold cuts (CC) and one strain isolated frozen food (FF): no. 469CC, 132FF, 470CC, 473CC and 472CC (Fig. 9). We observed significant differences between the clinical strains and cold cuts and frozen food groups (Fig. 9).

### Discussion

In 2020 *listeriosis,* associated with the consumption of food contaminated with *L. monocytogenes*, was the 5th zoonosis in the European Union, with a mortality rate of 13.0% [33]. High mortality, especially among the risk groups (the elderly, pregnant women, people in immunosuppression) determines the constant monitoring of *L. monocytogenes* and the correct design of the elimination strategies for this pathogen. The ability to survive and adapt to adverse conditions during host invasion and food processing, is a key aspect for hygiene strategies. A relevant problem is the ability to survive and multiply under refrigerated conditions, especially in RTE products that do not require prior heat treatment [34]. Many researches on the stress response of *L. monocytogenes* have been carried out on a small number of strains. As the population of *L. monocytogenes* is heterogenous, it is advantageous to study the stress response in a larger group of strains isolated from various sources. Our study evaluated 50 strains isolated from food (salmon, cold cuts, frozen food) and clinical material (including cases of systemic infections).

The PFGE method, referred to as the "gold standard", was used to assess the genetic similarity of the tested isolates. Currently the PFGE and WGS methods are most popular methods used for typing of microorganisms. The PFGE method is widely apllied for, e.g., outbreaks' investigation [35], food safety [22], identification of persistent strains [12, 23–25]. The advantages of the PFGE method include high discriminatory power, standardization of protocols and the availability of large databases for pathogen PFGE banding patterns. Unfortunately, this method is time-consuming and requires appropriate equipment [17]. Currently, the WGS method is used more and more often, especially for epidemiological purposes [36–39]. This method is more accurate than PFGE. Researchers have proven that some strains considered identical using the PFGE method, were in fact different [32]. Data obtained with WGS are highly reproducible. WGS allows identification of [32] virulence and antibiotic resistance determinants [32, 40]. The disadvantage of the WGS method is the large amount of information generated during the analysis, which is associated with the difficulty of storing digital data [41]. In our study, using the PFGE method, we identified 50 genetically different strains.

The initial characterization of the strains included the determination of serogroups belonging, the presence of virulence genes and the assessment of drug susceptibility. We have shown that most studied strains represented the serogroup 1/2a-3a (31 (62.0%)), followed by the serogroup 4b-4d-4e (14 (28.0%)). *L. monocytogenes* 4b, 1/2b and 1/2c serotypes are most often responsible for listeriosis (98% of documented cases) [42]. In contrast, serotype 1/2a and 1/2c isolates are more frequently isolated from food and environmental samples [43, 44]. In our study, 60.0% of the strains had all ten virulence genes tested. However, we confirmed the presence of LIPI-1 genes in 38 (76.0%) strains. Importantly, the *L. monocytogenes* infection cycle mainly involves genes located on LIPI-1 [45]. Due to increasing antimicrobial resistance in recent years, we also assessed the drug susceptibility of the strains. Among the studied population, as many as 92.0% of the strains were susceptible to 5 tested antibiotics. Three strains isolated from the clinical material were resistant to penicillin. Continuous monitoring of antibiotic resistance among isolates from different sources is necessary. In our study, we did not show the effect of antibiotic resistance on higher tolerance of stress factors. On the other hand, in the study by Komora et al. [5], antibiotic-resistant isolates were less susceptible to acid and osmotic stress.

Scientists are constantly searching for new methods of microbiological food control. The study assessed the effect of the bacteriophage in PhageGuard L on *L. monocytogenes*. The concentration of bacteriophage inhibiting the growth of the largest number (22; 44.0%) of the strains was 5 × 10^8^ PFU. In turn, Reinhard et al. [46] showed that PhageGuard L reduced *L. monocytogenes* from 1.27 to 3.33, 1.17 to 2.76, 1.19 to 1.76 log_10_ CFU/cm^2^ on the surface of stainless steel, polyurethane thermoplastic tape and on the epoxy resin floor, respectively. Additionally, Reinhard et al. [46] confirmed that higher phage concentration (10^8^ PFU/cm^2^) and longer contact time (3 h) resulted in a higher reduction of *L. monocytogenes*. Also Truchado et al. [47] have showed efficacy of PhageGuard L (working solution 10^9^ PFU/ml) against *L. monocytogenes*. They have observed maximum reduction of *L. monocytogenes* (by about 3.5 log units) on freshly cut endives after 3 days of storage [47]. Based on the presented results, the use of bacteriophages may be a promising treatment limiting the presence of *L. monocytogenes* on food products or food contact surfaces. However, more research on its safety and effectiveness is required.

The stress adaptive response of bacteria is one of the greatest challenges in controlling pathogens within the food industry. A relevant is balance between the microbiological safety of food and maintaining the freshness of products [48]. To date researchers have documented response of *L. monocytogenes* to many stress factors, including heat and cold shock, acids, high salt content and preservatives [49–54]. Our study assessed tolerance of 50 strains isolated from various sources to the stress factors associated with food processing and encountered during hostage invasions.

Since salt is a common food preservative, especially in meat and fish products, osmotic stress is one challenge in the food processing environment for *L. monocytogenes* [55]. The most often used concentration in brine, especially in smokehouses in the fish industry, is 3% NaCl. Contact with osmotic stress during the food processing may affect the later adaptation abilities of *L. monocytogenes*. The main mechanism of osmotic stress response in *L. monocytogenes* relies on osmoprotectants (e.g., glycine betaine and carnitine) [56]. *L. monocytogenes* is able to grow at high salt concentrations. Rods can withstand up to 10% of NaCl [57] and 12% (w/v) (30˚C) [58]. In our study, all strains tolerated up to 6% NaCl. The NaCl concentration of 7% inhibited the growth of 5 strains. In turn, 8% NaCl was enough to hinder multiplication of 31 strains (62.0%). One strain isolated from salmon (17S) withstood the NaCl concentration of 15%. The increased resistance of this strain may be related to earlier contact with the stressor in the fish plant or is an individual feature of the strain. On the other hand, Liu et al. [59] showed that all *L. monocytogenes* strains tested were resistant to saturated NaCl (about 7 M (40% w/v)) for a long time (20 h and possibly longer). In turn, Magalhães et al. [60] observed that persistent strains (isolated from cheese-processing) better adapted to growth in 2.5, 4, and 8% NaCl than sporadic strains. Patchett et al. [61] showed that at 5% NaCl, cells contained elevated levels of potassium betaine and glycine. At 7.5% of NaCl concentration of glycine-betaine, glycine, alanine and proline increased. In contrast, at 2.5% (w/v), there was no increase in accumulation of any of the solutes measured [61]. The temperature may also have an influence on the higher salt concentration tolerance [54, 58, 62]. Shahamat et al. [63] found that *L. monocytogenes* was able to survive in soybean tryptose broth containing 25.5% (w/v) NaCl for 4 days at 37°C, and lowering the experimental temperature to 22°C extended the survival time to 24 days and up to over 132 days at 4˚C. We conducted experiment at 37˚C and did not observe relevant differences in salt stress resistance between strains of diiferent origin and serogroup. Also, Hingston et al. [64] noted no significant differences between the growth rates of different serotypes in 6% NaCl. The response of *L. monocytogenes* to high concentrations of NaCl varies and may depend on the time-exposure, previous contact with the stressor (including the source of isolation), the availability of osmocrotectants in the environment, temperature or individual strain. It is also important to remember that exposure of *L. monocytogenes* to osmotic shock can contribute to cross-protection against other stresses such as heat and acid [65].

*L. monocytogenes* in the food industry are exposed to changing environmental pH conditions. Since the use of acids (benzoic, salicylic, lactic, and propionic acid) is a routine food preservation method, acid stress is a common phenomenon. Also, during host invasion *L. monocytogenes* encounters low pH, i.e., gastric juice and bile salts [66]. The main mechanisms of acid stress response in *L. monocytogenes* include acid tolerance response (ATR), glutamate decarboxylase (GAD), arginine deiminase (ADI), and F_1_F_0_-ATPase [67]. In our study we investigated the growth ability of bacterial strains in a wide range of pH. We found that pH 5 and pH 9 hindered the multiplication of 19 strains (38.0%) and 20 strains (40.0%), respectively. It is worth mentioning that strains inhibited at pH 5 were able to grow at high pH values (up to 10), which may indicate their better adaptation to alkaline conditions. Many researchers have reported the inhibitory effect of low pH and organic acids on *L. monocytogenes* [52, 68, 69]. Phan-Than et al. [70] revealed that pH 4 and 3.5 inhibited the growth of *L. monocytogenes* LO28 and EGD strains, respectively. Metselaar et al. [48] concluded that the mechanisms responsible for increased acid resistance (low pH) are most likely only related to survival during severe pH stress. Interestingly, prior exposure to non-lethal pH for several hours increases the ability to adapt to subsequent lower pH values [70–75]. Additionally bacteria better withstand acidic pH in rich media than in the minimal [71, 76]. Metselaar et al. [48] distinguished three groups of strains – group highly resistant to acid stress, group with slightly increased resistance and one isolate characterized by a variable reaction to pH. The increased acid resistance correlated with a decreased growth rate [48]. Cataldo et al. [77] demonstrated an enhanced response to acids and the maintenance of *L. monocytogenes* invasiveness after exposure to acid stress. Researchers have shown [76, 77] that resistance to acid stress in *L. monocytogenes* is possibly associated with the synthesis of appropriate proteins that change the structure of the cell membrane. As a result bacterial ability to maintain intracellular pH increases. It suggests that to different pathways for H^+^ entry and exit through the cell membrane. In response to lethal acidic conditions bacteria synthesize additional stress proteins [70, 78, 79]. Weak organic acids are more detrimental to *L. monocytogenes* than strong inorganic acids such as HCl [70]. In the current study, we used HCl and NaOH to change the broth pH. Our study showed no statistically significant differences in response to changing pH between different strains’ groups. In contrast, Dykes and Moorhead [80] found lower log CFU values after exposure to pH 2.5 (2 h) for strains isolated from meat. Vialette et al. [81] showed that clinical strains better responded to acid stress than environmental strains. Also, Komora et al. [5] have shown a statistically lower logarithmic reduction numbers s after acid and osmotic stress in clinical strains than in food strains. Barbosa et al. [82] and Ramalheira et al. [83] have assessed the behavior of *L. monocytogenes* during passage through the simulated gastrointestinal tract. They have observed that clinical isolates were more resistant than food isolates recovered from various food products. On the other hand, Lundẻn et al. [84] have noted that persistent strains showed higher tolerance to acidic conditions than sporadic strains. Giotis et al. [58] have documented that exposure to alkaline stress (pH above 9) affected the morphology of *L. monocytogenes* cells. Such atypical cells may be associated with increased survival of *L. monocytogenes* in unfavorable environments [85]. Taormina and Beuchat [86] have reported that *L. monocytogenes* cells survived for at least 6 days in tryptose phosphate broth at pH 9.0, 10.0, and 11.0 at 4°C or 21°C. *L. monocytogenes* may also experience alkaline stress in the food processing environment. Alkaline solutions are generally used in detergents to remove charred sediment, oil or grease, facilitate protein denaturation, saponification of fats. Interestingly, our own study showed that 12 strains isolated from clinical material multiplied at pH 10. Many factors have an impact on the ability to survive and grow at different pH, including the individual characteristics of the strain.

Application of low and high temperatures is a well-known and widely used method of eliminating microorganisms. *L. monocytogenes* have the ability to grow in a wide range of temperatures (0–45˚C) [87]. We observed the growth inhibition afer 30-min exposure at 65˚C. Strains isolated from the clinical material were more heat-resistant than other strains. Five strains (4 isolated from clinical material, and one isolated from salmon) survived the 60-min exposure to 70˚C. On the other hand, Vasseur et al. [54], after heat shock, have observed different growth delay times. A study by Komora et al. [5] has demonstrated no differences in the survival between different *L. monocytogenes* strains subjected to heat stress (temperature 58˚C). Hingston et al. [64] found no clear trends in response to heat stress conditions related to the origin of isolates (food-sourced versus clinical isolates). In turn, Shen et al. [88] divided the strains into three heat stress response groups, low (< 2 log CFU/ml), medium (2 to 4 log CFU/ml) and high (4 to 6 log CFU/ml) resistance. Sub-lethal heat stress at 48˚C for 60 or 90 min increased *L. monocytogenes* lag phase delay in trypic soy broth supplemented with 0.6% yeast extract at room temperature for 3 to 5 h compared to non-stressed control cells [88]. The strains of serotype 1/2a had relatively low heat tolerance [88]. Therefore, the serotype may not be the only factor influencing heat tolerance.

The ability of *L. monocytogenes* to grow under refrigerated conditions, transport or refrigerated food storage by the consumer increases the risk of infection or epidemics. A number of researchers have studied the cold stress response of *L. monocytogenes* [89–92]. Our study showed the survival and growth of all *L. monocytogenes* at 4˚C. After 30 days of incubation at 4˚C clinical strains reached the highest number (increase by 4.05 log CFU). The final total bacteria numbers were 10.12, 10.42 and 10.12 log CFU for strains isolated from frozen food, salmon and cold cuts, respectively. However, the differences between groups were not significant. Arguedas-Villa et al. [93] have demonstrated no correlation between the cold stress response and the serotype or genetic line. In turn, Vasseur et al. [54] have found that cold stress did not affect the growth of the studied strains. We also did not notice the link between cold growth and source of isolation.

Freezing food is a process that extends the shelf life of a product and is also readily used by consumers. Freezing and thawing are big challenges for bacteria, including *L. monocytogenes* [94]. Yoon et al. [95] have found that freezing of *L. monocytogenes* for 5 days resulted in an extension of the lag time and a slowdown in the specific growth rate. In turn, Miladi et al. [96] have observed a reduction (by 3.69 log) of *L. monocytogenes* cells stored for 10 months at -20˚C. The researchers found no changes in the metabolism of *L. monocytogenes* [96]. Also Ben Slama et al. [97] have noted the survival of *L. monocytogenes* after six months of freezing (-20˚C) on cheese slices. Jiang et al. [98] showed that the freezing procedure significantly enhanced the effect of pectin-based anti-*Listeria* coatings, reducing the population of *L. monocytogenes* on roasted turkey. The research conducted so far, has focused on the survival of *L. monocytogenes* mostly under freezing conditions. Our study assessed the impact of cyclic freezing and defrosting, which is essential from the consumer's point of view. In our study, we demonstarted that the bacteria number after one cycle of freezing was significantly higher than the control group (not subjected to freezing). Strains isolated from clinical material, cold cuts and frozen food increased their number up to the third freezing-defrosting cycle. After the fourth cycle, all bacteria significantly decreased their number. In the defrosting variant, only after the 4th cycle we noticed an increase in log CFU compared to the control. In contrast, Azizoglu et al. [99] have shown marked cryotolerance of *L. monocytogenes* cells grown at 37°C, with a decrease of < 1 log after 18 freeze–thaw cycles. Conversely, freeze–thaw tolerance was significantly reduced when bacteria were grown at 4 or 25°C (2 to > 4 log). On the other hand, Simpson Beauchamp et al. [100] have documented that thawing treatments had little effect on *L. monocytogenes* populations (< 0.5 log CFU/cm (2)). In turn, Kang et al. [101] have observed that freezing cold smoked salmon prior to bacterial inoculation significantly increased *L. monocytogenes* number at 7°C. Due to a significant gap in the data on the effect of freezing–thawing on *L. monocytogenes* further studies are recommended.

The availability of nutrients in the environment varies. The food processing environment is rich in waste, such as blood, fruit juices and food fragments. The availability of nutrients decreases significantly after disinfection procedure. *L. monocytogenes* can grow and multiply in environments with varying nutrient availability. In our study, in conditions of low nutrient content, strains isolated from frozen food performed best (visible increase in log CFU number), which may be associated with previous freezing. High BHI content favored the growth of strains of all groups. In the environment of insufficient nutrients availability *L. monocytogenes* initiates the starvation survival response (SSR) [102]. Several researchers [80, 103, 104] have presented data on the mechanisms of survival in conditions of glucose deficiency. Importantly, Herbert and Foster [104] have shown that SSR in *L. monocytogenes* EGD can be induced by glucose and other nutrients restriction but not amino acid restriction. Surviving cells show reduced cell size and increased cross-protection to several environmental stresses [102, 104]. Attempts have been made to explain the SSR phenomenon. The initiation but not the maintenance of an SSR involves both protein and cell wall biosynthesis. It is also likely that the nutrients released from the dead cells are recycled to allow the remaining population to survive [104]. Lungu et al. [105] suggest that the SSR of complete nutrient starvation in the presence or absence of oxygen is independent of the SigB. However, the mechanisms governing the SSR of *L. monocytogenes* during multicomponent starvation are still not fully understood.

In our ranking of the stress response, four clinical strains and one isolated from salmon got the highest score. Clinical strains were more resistant to the stress factors used in the study. Clinical strains derived from, e.g., blood or cerebrospinal fluid, which may indicate their high virulent potential. Nevertheless, the response of food strains was heterogeneous. Strains isolated from cold cuts received the lowest number of points in the ranking (103.5 points). It may support Dykes and Moorhead [80] suggestions that only some strains of *L. monocytogenes* found in food products exhibit full pathogenic potential. High adaptability to unfavorable stress conditions may increase microbial virulence [106]. Kazmierczak et al. [107] have reported that stress exposure of *L. monocytogenes* is associated with a higher expression of virulence genes (including internalin) [107].

### Conclusions

*L. monocytogenes* is one of the most dangerous foodborne pathogen. Its ability to survive and grow under various stressful conditions in the food production environment, is of great concern. Knowledge about the influence of stress factors on *L. monocytogenes* will allow to design appropriate methods of control and production line disinfection. Adaptation to stress factors is an individual feature of a given strain, and so far no clear link between stress response and the strains' origin has been described. We included, in our study, clinical and food strains to have a more diverse population of *L. monocytogenes*. We expect that some strains could have experienced earlier adverse conditions. Nonetheless, evaluation of the impact of stress factors on phenotypic traits and virulence of *L. monocytogenes* merits further investigation. Our study aimed to screen a stress response to factors most commonly encoutered in the food production environment in large population. Future studies should concentrate on the molecular bases of the observed resistance to stress factors.

### Materials and methods

#### Material

The research material consisted of 80 *L. monocytogenes* isolates from the collection of the Department of Microbiology, Ludwik Rydygier Collegium Medicum in Bydgoszcz, Nicolaus Copernicus University in Toruń, Poland, previously isolated from clinical material (C—clinical, *n* = 20) and food (*n* = 60 (S – salmon (*n* = 20), CC – cold cuts (*n* = 20), FF – frozen food (*n* = 20)) (Table 6). The belonging to serological groups, the presence of virulence genes, drug susceptibility of clinical strains was assessed earlier by Dr. Katarzyna Grudlewska-Buda (unpublished data). The influence of selected stress factors on these strains was the subject of these research. Table 6 presents the origin of the clinical strains. The clinical strains come from the Department of Microbiology, Ludwik Rydygier Collegium Medicum in Bydgoszcz, Nicolaus Copernicus University in Toruń, Poland. The strain ID used preclude the identification of the patient from whom the *L. monocytogenes* strain was isolated. The reference strain *Listeria monocytogenes* ATCC 19111 was used in the experiment. The all isolates were stored in brain–heart infusion broth (BHI, Merck) with 15.0% glycerol (Avantor) at − 80°C until the beginning of the research.

**Table 6:** Origin of *L. monocytogenes* isolates used in the study

Origin	Food isolates (*n* = 60; 75.0%)			Clinical isolates (*n* = 20; 25.0%)
	Salmon (*n* = 20; 25.0%)	Cold cuts (*n* = 20; 25.0%)	Frozen food (*n* = 20; 25.0%)	•blood (*n* = 4; 5.0%) •cerebrospinal fluid (*n* = 4; 5.0%) •vaginal swab (*n* = 4; 5.0%) •cerrical swab (*n* = 1; 1.0%) •ear swab (*n* = 1; 1.0%) •pharynheal swab (*n* = 1; 1.0%) •descendant tumor (*n* = 1; 1.0%) •heat valve (*n* = 1; 1.0%) •blood from the catheter (*n* = 1; 1.0%) •peritoneal fluid (*n* = 1; 1.0%) •dialysis fluid (*n* = 1; 1.0%)

### Preparation of *L. monocytogenes* for research

Tested isolates were plated on Columbia Agar supplemented with 5.0% sheep blood (CAB, Graso) and incubated (37°C, 24 h). Then, the grown colonies were cultured under the same conditions. The grown strains of *L. monocytogenes* were used for further studies.

### Evaluation of genetic similarity

After confirming the species identity, the genetic similarity of the selected *L. monocytogenes* isolates was determined with PFGE (“gold standard”). The procedure for genotyping was performed in accordance with the standard operating procedure for PulseNet PFGE of *Listeria monocytogenes* [108]. To determine the degree of genetic similarity between isolates, a phylogenetic dendrogram was drawn in the CLIQS 1D Pro program (TotalLab). Clustering analysis was performed using hierarchical clustering with the UPGMA technique and Dice’s coefficient. The PFGE technique was the only method of evaluating the genetic similarity of the isolates tested.

### Preliminary characterization of strains

The initial characterization of *L. monocytogenes* strains included the assessment of belonging to serological groups, the presence of specific virulence genes and sensitivity to selected antibiotics. The presence of LIPI-1 which contains virulence genes involved in the intracellular infection cycle of *L. monocytogenes* and locusInlA-InlB was also assessed.

#### Isolation of genomic DNA

DNA was isolated from overnight cultures grown on Tryptic Soy Agar (TSA, Graso). A single colony was resuspended in 100 µl of 1 × Tris–EDTA buffer (Sigma-Aldrich), heated at 90°C (theromblock, Eppendorf) for 10 min, cooled on ice (2 min), and centrifuged (16,000 × g, 5 min).

### Molecular serotyping of *L. monocytogenes*

Multiplex PCR for the identification of the main *L. monocytogenes* serogroups (1/2a-3a, 1/2b-3b, 1/2c-3c, and 4b-4d-4e) was performed as described by Doumith et al. [109]. The four selected *L. monocytogenes* strains described earlier by Wałecka-Zacharska et al. [110] were used as control strains for serogroups identification.

#### Detection of selected virulence genes

The multiplex PCR technique was used to determine the frequency of 10 selected virulence genes occurrence among *L. monocytogenes*. Three separate PCR reactions were prepared: I (genes: *fbpA*, *plcA*, *hlyA*), II (genes: *plcB*, *inlB*, *actA*, *iap*), and III (genes: *inlA*, *mpl, prfA*). Primer sequences and reaction conditions were previously presented by Skowron et al. [111]. The multiplex PCR reactions were set using the previously isolated genomic DNA. The *L. monocytogenes* ATCC 19111 strain, possessing all detected genes, was used as the reference.

#### Evaluation of drug resistance

The evaluation was performed using the disk-diffusion method. Bacterial cultures (24 h) were diluted to 0.5 McF in 0.9% saline solution (Avantor). The prepared suspensions were plated on MHF medium (Mueller Hinton Agar with 5.0% horse blood and 20 mg/L β-NAD, Graso) and then antibiotic discs were added. The susceptibility of isolates to penicillin (1 IU), ampicillin (2 μg), meropenem (10 μg), erythromycin (15 μg), and cotrimoxazole (1.25–23.75 μg) was evaluated. The prepared antibiograms were incubated at 35°C for 20 h. Then, growth inhibition zones around the antibiotic discs were measured. The results were analyzed in accordance with the EUCAST (The European Committee on Antimicrobial Susceptibility Testing) v. 12.0 recommendations [112].

### Evaluation of Minimum Inhibitory Concentration (MIC) and Minimum Bactericidal Concentration (MBC) of Phage Guard L

The evaluation was carried out on the basis of Vipra et al. [113], with some modifications. 24-h bacterial cultures were diluted to 0.5 McF in tryptic soy broth (TSB, Becton Dickinson). The suspension was diluted according to EUCAST recommendations (final concentration: 5 × 10^5^ CFU/mL) [112]. Then, 100 µl of the bacterial suspension was introduced into a multi-well plate (in triplicate). 100 µl of Phage Guard L (Micreos Food Safety) bacteriophage at the appropriate concentration was added to the bacterial suspension (final concentration 5 × 10^4^ to 5 × 10^10^ PFU). The negative control was 100 µl of bacteriophage of appropriate concentration and 100 µl of sterile TSB. The positive control consisted of 200 µl diluted bacterial suspensions. Incubation was carried out in a moist chamber (35°C, 20 h). Next, the turbidity was assessed visually. Turbidity indicated the growth, and no turbidity marked the growth inhibition of *L. monocytogenes* (MIC value). For the identified MIC value concentration and the three above concentrations of bacteriophage suspension, bacteria were streaked on the TSA medium to determine the MBC value. After 24-h incubation at 37°C the growth was assessed (MBC value). No growth on the medium after the incubation period was taken as the MBC value.

### Influence of selected stress factors on the growth and survival of *L. monocytogenes*

#### Osmotic stress

The final NaCl concentration used for the experiment ranged from 0 to 20% (variation every 1%). Suspensions of the tested strains in Mueller Hinton Broth (MHB, Becton Dickinson) (0.5 McF) were mixed with an equal volume of MHB with the appropriate NaCl concentration (in triplicate). The test included a positive control (suspension of the given strain) and a negative control (sterile MHB). After the incubation period (24 h, 37˚C) in a moist chamber, the presence of turbidity was assessed visually (screenig method). Turbidity indicated the growth, and no turbidity marked the growth inhibition of *L. monocytogenes.*

#### Acid and alkaline stress

The pH tested ranged from 3 to10 (variation every 1 degree). Suspensions of the tested strains in Mueller Hinton Broth (MHB, Becton Dickinson) (0.5 McF) were mixed with an equal volume of MHB with an equal volume of MHB at the appropriate pH (using HCl (POCH) or NaOH (Chempur)) (in triplicate). The test included a positive control (suspension of the given strain) and a negative control (sterile MHB). After the incubation period (24 h, 37˚C) in a moist chamber, the presence of turbidity was assessed visually (screening method). Turbidity indicated the growth, and no turbidity marked the growth inhibition of *L. monocytogenes.*

#### Heat and cold stress

The impact of high temperatures, the so-called heat shock (55, 60, 65 and 70˚C) and low temperature, the so-called cold shock (4˚C), on the survival rate of *L. monocytogenes* were assessed. A suspension of strains in Phosphate Buffered Saline (PBS, BTL) (0.5 McF) was subjected to selected temperatures for an appropriate time (heat shock: 2, 15, 30 and 60 min; cold shock 24 h, 3, 10, 15, 30 days). Then, a serial tenfold dilutions in PBS (BTL) were prepared and plated (100 µl) on sheep blood agar (CAB, Columbia Blood Agar, Graso). After the incubation period (24 h, 37˚C), grown colonies were counted and presented as log CFU/ml. The negative control was sterile PBS, and the positive control was a suspension of the tested strains.

#### Effect of cyclic freezing (-20˚C) and defrosting

A suspension made in Tryptic Soy Broth (TSB, Graso) (0.5 McF) was placed at -20°C for one day. Next, the samples were defrosted at 23°C for three hours (complete liquefaction) and serial tenfold dilutions were plated (100 µl) on CAB (Graso) medium (in duplicate). After the incubation period (37°C, 24 h), the grown colonies were counted and presented as log CFU/ml. The sample was then placed back in the freezer. The procedure was repeated three times. A sample not subjected to cyclic freeze-defrosting (frozen, stored at -20°C and defrosted in the last cycle of the experiment) was also prepared.

#### The influence of nutrients

A broth with a weight of loose substrate ranging from 20 to 400% recommended by the manufacturer was used (variation every 20%). Strain suspensions in Brain Heart Infusion (BHI (Becton Dckinson) (0.5 McF) were diluted 500-fold. Then 100 µl of such suspension was mixed with 100 µl of BHI medium (Becton Dickinson), obtaining 10—200% availability of nutrients. A positive control (suspension of the given strain) and a negative control (sterile BHI) were prepared. After the incubation period (37°C, 24 h) serial tenfold dilutions were made and 100 µl was plated onto CAB (Graso) (in duplicate). After the incubation period (24 h, 37˚C), grown colonies were counted and presented as log CFU/ml.

### Statistical analysis

All statistical analyses were performed using R software [114]. Due to absence of a normal distribution (as verified with the Shapiro–Wilk test), all continuous variables were analyzed using non-parametric tests. The primary variable for this study was change in the number of bacteria expressed as the logarithmic value of colony forming unit (CFU) per ml (if applicable). Differences between groups (due to the source of origin (clinical, salmon, cold cuts, frozen food)) were assessed using the Kruskal–Wallis test followed by the Bonferroni Dunn multiple comparison test. For repeated measurements, paired Wilcoxon signed-rank test with Bonferroni correction was used. For categorical data counts with percentages were used and the Fisher’s exact test was performed. A two-sided p-value equal or less than 0.05 was considered significant.

In order to select the most resistant strains, a scoring system based on non-parametric rank approach was used. The strains were ranked based on their susceptibility to each of the five stresses in a following manner:

- (a) osmotic stress – based on saline concentration that inhibited the growth of the strain (the higher concentration – the higher rank);
- (b) pH range – based on range at which the growth of the strain was not inhibited (the higher range – the higher rank);
- (c) heat stress—based on values of log CFU/ml (expressed as change of the number of bacteria after stress exposure); since there were multiple conditions (four time points and four temperatures), mean ranks for each time point/temperature combination were calculated to obtain the final rank;
- (d) high or (e) low nutrient access – also based on values of log CFU/ml; mean ranks for each nutrient availability level (low: 10–40%, high: 130–200%) were calculated to obtain final rank.

The strains could score a maximum of 50 points for each stress (the maximum value is based on the number of strains), therefore, in total, there were 250 points to score. In the case of ties, the average score was calculated. Strains with the highest scores were considered the most resistant. A limitation of this approach is that some of the variables were expressed in a discrete scale, resulting in multiple ties.

## Data Availability

The datasets used and/or analysed during the current study available from the corresponding author on reasonable request.

## Abbreviations

R: Resistance
S: Sensitive
P: Penicillin
AMP: Ampicillin
MEM: Meropenem
E: Erythromycin
STX: Co-trimoxazole (sulfamethoxazole + trimethoprim)
MIC: Minimum inhibitory concentration
MBC: Minimum bactericidal concentration
PFU: Plaque forming units
MHF medium: Mueller Hinton Agar with 5.0% horse blood and 20 mg/L β-NAD
PFGE: Pulsed-field gel electrophoresis
EUCAST: The European Committee on Antimicrobial Susceptibility Testing
MHB: Mueller Hinton Broth
PBS: Phosphate Buffered Saline
TSB: Tryptic Soy Broth
RTE: Ready-to-eat
FPE: Food processing environment
C: Clinical
CC: Cold cuts
S: Salmon
FF: Frozen food
BHI: Brain Heart Infusion
ATR: Acid tolerance response
GAD: Glutamate decarboxylase
ADI: Arginine deiminase
CFU: Colony forming units
CAB: Columbia agar supplemented with 5.0% sheep blood
TSA: Tryptic Soy Agar
PCR: Polymerase chain reaction
ATCC: American Type Culture Coletion
SSR: Starvation survival response
RAPD: Random Amplified Polymorphic DNA
RFLP: Restriction Fragments Length Polymorphism
AFLP: Amplified Fragment Length Polymorphism
WGS: Whole genome sequencing
MALDI TOF MS: Matrix-assisted laser desorption/ionization time of flight mass spectrometry
MLST: Multi-Locus Sequence Typing
LIPI-1: Listeria Pathogenicity Island 1
